# Perception About Transplant of Rural and Urban Patients With Chronic Kidney Disease; A Qualitative Study

**DOI:** 10.5812/numonthly.15726

**Published:** 2014-03-02

**Authors:** Nasrollah Ghahramani, Chloe Wang, Ali Sanati-Mehrizy, Ankita Tandon

**Affiliations:** 1 Department of Medicine, Pennsylvania State University College of Medicine, Hershey, USA; 2 Pennsylvania State University College of Medicine, Hershey, USA

**Keywords:** Focus Groups, Geography, Disparity

## Abstract

**Background:**

Chronic kidney disease (CKD) is a worldwide public health problem with increasing incidence and prevalence and associated expenses.

**Objectives:**

To explore different perceptions of rural and urban patients with chronic kidney disease (CKD) about kidney transplant.

**Patients and Methods:**

We conducted four focus groups, each including 5 or 6 patients with stage 5 CKD or end stage renal disease living in a rural or urban area. Open-ended questions probed patient familiarity with kidney transplant, perceptions of benefits of kidney transplant, perceived barriers to kidney transplant, and views about living donation. All the sessions were recorded and professionally transcribed. Responses were pooled, de-identified, and analyzed using qualitative thematic content analysis.

**Results:**

Urban patients were more likely to receive supplementary information and being strongly encouraged by their nephrologists to seek transplant. All participants acknowledged “independence” as the main advantage of transplantation. Increased freedom to travel and improved life expectancy were mentioned only among the urban groups. The main themes in all groups regarding perceived barriers to transplant were the tedious pre-transplant testing and workup expenses. Among rural groups, there was a perception that distance from transplant centers impedes transplant evaluation. Religious reasons favoring and opposing transplant were mentioned by participants in a rural group. Some members contended that since illness is God’s will, we should not change it. Others in the same group argued that “God is not ready for us to give up”. Praise and gratitude for the living donor were expressed in all groups, but concerns about donor’s outcome were discussed only within the rural groups. In discussing preference about known or anonymous donors, members of an urban group mentioned favoring an anonymous donor, citing unease with a sense of life-long indebtedness.

**Conclusions:**

Observed differences in perceptions among rural and urban patients about aspects of transplant may contribute to geographic disparities in transplant. The findings could be helpful to guide future individualized, culturally sensitive educational interventions about transplant for patients with CKD.

## 1. Background

Chronic kidney disease (CKD) is a worldwide public health problem with increasing incidence and prevalence and associated expenses (1, 2). The most accepted treatment for end stage renal disease (ESRD) is in-center hemodialysis worldwide (3, 4). Transplant leads to improved survival, a better quality of life, and lower costs compared to long-term dialysis. It is the treatment of choice for most patients (5, 6). Among transplant options, pre-emptive living donor transplant is associated with better graft and patient survival (7). Despite well-known advantages of renal transplant, there is a wide geographic variation in the rate of kidney transplant (8, 9). Patients living in rural regions are up to 15% less likely to be placed on the waiting list (10). The contribution of patient attitudes and perceptions to geographic disparities in transplant rates is not well defined. We hypothesize that urban-rural differences in transplant rates reflect differences in perceptions of patients about transplant.

## 2. Objectives

Our aim was to explore rural-urban differences in perception about kidney transplant among patients with CKD. Specifically, we investigated four themes, which included familiarity with transplant, perceptions of benefits of transplant, perceived barriers to kidney transplant, and views about living donation among patients with CKD.

## 3. Patients and Methods

### 3.1. Study Design and Recruitment of Participants

The Institutional Review Board of Penn State College of Medicine approved the research protocol. The protocols conformed to the guidelines of the 1975 Helsinki Declaration. Written consent forms were completed by each subject. Four 2-hour focus group sessions were planned to have two focus group sessions each for patients living in urban and rural areas, respectively. Eligible participants were adult patients with stage five CKD or ESRD, willing to participate in group discussion and physically able to attend focus group meetings. We recruited participants by posting flyers in dialysis units and nephrologists’ offices in the central region of Pennsylvania. The flyers described the purpose of the study, the intended duration, date, time, and location of the sessions, and the honoraria provided for participation. Those interested were asked to directly contact the principal investigator to obtain further details. We recruited 10 participants for each focus group to ensure a group of at least five participants. Participants were assigned to one of four focus groups depending on their home address either a rural or an urban area, based on rural-urban commuting area codes, and whether they preferred a morning or an afternoon meeting. The principal investigator contacted each volunteer by telephone one day before the group meeting to confirm his or her participation.

### 3.2. Topic Guide

We developed a topic guide based on literature review and peer discussion. The guide was revised following pilot testing among seven dialysis patients at the principal investigator’s institution. Key questions consisted of broadly stated open-ended queries probing the participants’ familiarity with transplant, perceptions of benefits of transplant, perceived barriers to transplant, and views about living donation.

### 3.3. Data Collection

The principal investigator initiated each session by introducing himself and asking participants to introduce themselves, with the option of using an alias. He then described the research and answered questions. The participants were reassured of the confidentiality of their comments and were also reassured that all collected information, including audio-recordings of the sessions would be anonymous and eliminated after completion of data analysis. The participants were informed about their right to withdraw from the study at any point. Each participant then completed the informed consent, including consent to audio recording and transcription of the discussion and completed an anonymous demographic questionnaire. For consistency, the principal investigator moderated all sessions using reflective probes for clarification of statements. To ensure balanced participation and to avoid domination by any group member, we actively encouraged all participants to express their views (11). This approach minimizes the potential for acquiescence bias, in which the group members tend to conform to the dominant view (12). All sessions were audio recorded and transcribed verbatim by a professional transcriptionist.

### 3.4. Data Analysis

Quality review of each session’s transcript was conducted. Two reviewers independently reviewed each transcript to identify recurrent response themes. Either the group or the individual may be considered for data analysis of qualitative studies (13). In our study, we de-identified pooled responses from individuals within each category of respondents (urban and rural). We then used qualitative thematic content analysis (14, 15), by thoroughly searching the transcripts to identify dominant and recurrent themes.

## 4. Results

Four separate focus groups were conducted with 23 participants. Each focus group consisted of five or six patients with CKD living in rural (n = 11) or urban area (n = 12). Demographic characteristics of the participants are outlined in Table 1. The dominant themes and sub-themes emerged during focus group discussions of related topics are outlined below and in Table 2. Urban participants were more likely to have received supplementary information, such as videos or pamphlets, from their nephrologists about transplant. Urban participants were also more likely to being strongly encouraged by their nephrologists to seek transplant. Both urban and rural participants acknowledged “more independence” as the main advantage of kidney transplant compared with dialysis. “Increased freedom to travel” and “improved life expectancy” were mentioned only among the urban participants as advantages. When exploring some of the perceived barriers about transplant, both urban and rural groups noted the tedious pre-transplant testing processes and workup expenses. Rural groups also noted the distance from transplant center as an additional impediment to transplant evaluation. Religious reasons favoring or opposing transplant were mentioned by participants in one of the rural focus groups. Some members contended that since “illness is God’s will, we should not change it”. Other members in the same group argued, “God is not ready for us to give up”. Praise and gratitude for the living donor were expressed in all groups; but concerns about donor’s outcome were discussed only within the two rural groups. In discussing preference about known or anonymous donors, members of one of the urban focus groups mentioned that they would prefer an anonymous donor, citing the unease with a sense of life-long indebtedness to the organ donor.

**Table 1.** Characteristics of the Focus Groups Participants (n = 23) ^a^

	Rural		Urban	
	Group 1 (n = 6)	Group 2 (n = 5)	Group 3 (n = 6)	Group 4 (n = 6)
**Female**	2	3	3	3
**Caucasian**	5	4	3	5
**Some college**	1	1	3	1
**On dialysis**	5	3	5	4
**Referred for Transplant**	3	2	3	2
**Listed for transplant**	2	1	2	2
**Age, Mean ± SD**	61 ± 15.6	65 ± 9.5	65 ± 9.9	59 ± 16.5
	63 ± 12.1		62 ± 13.1	

^a^ No significant difference.

**Table 2.** Themes and Sub-themes Identified According to Focus Group Characteristic ^a^

Themes	Sub-themes	
	Rural	Urban
**Familiarity with KT**		received supplemental info (video, pamphlet)
**Encouraged by nephrologists to pursue KT**		strongly encouraged
**Main advantage of KT vs. dialysis**	independence	independence
		freedom to travel
		improved life expectancy
**Perceived barriers to KT**	tedious testing process and cost	tedious testing process and cost
	distance from center	
	religious reasons opposing KT	
**Perceptions about living donation**	praise and gratitude for the donor	praise and gratitude for the donor
	concern about donor outcome	prefer anonymous donor (unease with sense of life-long indebtedness to known donor)

^a^ Abbreviation: KT, kidney transplant.

## 5. Discussion

This qualitative study yielded valuable insight into some of the factors affecting patient decisions about transplant. Comparing urban and rural patients with CKD, we found that both groups considered independence as the main advantage of kidney transplant, and tedious testing and cost as the major barrier to transplant and praise and gratitude for the donor. We also identified differences in perceptions of rural and urban patients about advantages of kidney transplant, barriers to transplant and views about living donation. Urban patients provided more details about the advantage of kidney transplant compared with dialysis, citing freedom to travel and improved life expectancy. This finding is consistent with the discussion among urban patients who received supplemental information about transplant and encouragement from their nephrologists to pursue transplant. It is not surprising that rural patients were more likely to mention distance from the transplant center as a major barrier to transplant.

The fact that concern about donor outcome was discussed in both rural groups but in none of the urban groups was interesting. It was also interesting that urban patients, given the option, noted preference for anonymous donor to someone known to them, expressing unease with the sense of life-long indebtedness. These two findings are congruent with suggestions and findings in the social sciences literature that life in rural areas is characterized by values that emphasize reciprocity and prioritize social obligation and duty. Adaptation to urban life, on the other hand, is associated with progressive individualism and self-prioritization (16-18). Religion influence on views about transplant was mentioned only among one of the rural groups. The role of religion in decision making related to health care among the rural population has been previously described (19). The main limitation of our study was selection bias. It is likely that, compared with other regions and countries, patients from central Pennsylvania have different views and perceptions about transplant. However, the principle conclusion of the study is the need to consider diverse perceptions and concerns about transplant, based on geographic regions. Concern about the notes and audio-taping may have potentially led to reluctance to participate. To overcome this reluctance, the moderator reviewed the purpose of the notes and recordings and explained procedures for protecting and handling confidential information to reassure the participants of confidentiality. Another potential limitation of our study was the moderator bias. The personal views and biases of the moderator, a transplant nephrologist, might have dominated the course of discussion (20). To minimize the moderator bias, we conducted semi-structured sessions, by using open-ended questions, which had been modified by pilot-testing.

We conclude that physicians should be aware of beliefs, concerns and fears of patients in discussing the option of transplant. The themes identified in this qualitative study would facilitate the development of quantitative studies of geographic variations in patient perceptions about transplant. The findings could be helpful to guide future individualized, culturally sensitive educational interventions about transplant for patients with CKD.
